# 4455 Advancing the Science of Community Engagement with Human-Centered Design

**DOI:** 10.1017/cts.2020.252

**Published:** 2020-07-29

**Authors:** Jordan Poll, Ayse Buyktur, Aalap Doshi, Linde Huang, Tricia Piechowski, Meghan Spiroff, Erica Marsh

**Affiliations:** 1 University of Michigan School of Medicine; 2 Michigan Institute for Clinical & Health Research

## Abstract

OBJECTIVES/GOALS: To describe how the Community Engagement (CE) Program at the Michigan Institute for Clinical & Health Research (MICHR), a Clinical & Translational Science Award (CTSA) site at the University of Michigan, is adopting human-centered design (HCD) to advance the science of community engagement in translational research and CE’s programmatic efforts. METHODS/STUDY POPULATION: The MICHR CE Program supports academic-community partnerships to transform translational research across the state of Michigan. As the team aims to better engage partners to help guide the direction of their work, CE is collaborating with MICHR’s Design and Innovation Core to incorporate human-centered design (HCD). HCD is an approach that prioritizes the needs, values, and perspectives of direct users during the creation of a new product or service. The MICHR team created interactive HCD activities for two statewide retreats to elicit feedback from community and academic members on ways to enhance community-engaged research (CEnR). Retreat participants worked on a variety of problems, such as barriers to partnering and defining impact for CEnR. These activities generated authentic, contextual, and multi-view data captured in various artifacts for systematic analysis. RESULTS/ANTICIPATED RESULTS: In the first retreat, a HCD activity had participants reflect on their own barriers to partnering in research and potential solutions. In the second retreat, an HCD activity facilitated participants interviewing each other on their views of the impact in CEnR. Results from the first activity identified a set of common barriers to CEnR, some related to partnership formation, communication, and partner equity, among others. These led the CE Program to specific programmatic efforts, such as designing a statewide partnership platform, hiring a communication program manager, and sponsoring community partners to join national conferences. The second retreat activity produced rich data to identify overlaps between different perspectives to inform how impact can be defined and measured in CEnR. DISCUSSION/SIGNIFICANCE OF IMPACT: HCD activities provide means to include community and academic members in the science of CEnR. They allow systematic ways to gather information directly from the diverse set of current or prospective partners of community engagement programs about their needs, experiences, and values, which can be translated to programmatic innovation.

